# A bibliometric analysis of electroencephalogram research in stroke: current trends and future directions

**DOI:** 10.3389/fneur.2025.1539736

**Published:** 2025-04-28

**Authors:** Xiao-Yu Liao, Yu-Er Jiang, Ren-Jie Xu, Ting-Ting Qian, Shi-Lu Liu, Yi Che

**Affiliations:** ^1^Department of Rehabilitation Medicine, The Affiliated Suzhou Hospital of Nanjing Medical University, Suzhou, China; ^2^Center for Excellence in Brain Science and Intelligence Technology, Institute of Neuroscience, Chinese Academy of Sciences, Shanghai, China; ^3^Department of Rehabilitation Medicine, Kunshan Rehabilitation Hospital, Suzhou, China; ^4^College School of Acupuncture-Moxibustion and Tuina, School of Health Preservation and Rehabilitation, Nanjing University of Chinese Medicine, Nanjing, China

**Keywords:** stroke, electroencephalogram, bibliometrix, CiteSpace, VOSviewer, visualization analysis

## Abstract

**Background:**

Electroencephalography (EEG) has become an indispensable tool in stroke research for real-time monitoring of neural activity, prognosis prediction, and rehabilitation support. In recent decades, EEG applications in stroke research have expanded, particularly in areas like brain-computer interfaces (BCI) and neurofeedback for motor recovery. However, a comprehensive analysis of research trends in this domain is currently unavailable.

**Methods:**

The study collected data from the Web of Science Core Collection database, selecting publications related to stroke and EEG from 2005 to 2024. Visual analysis tools such as VOSviewer and CiteSpace were utilized to build knowledge maps of the research field, analyzing the distribution of publications, authors, institutions, journals, and collaboration networks. Additionally, co-occurrence, clustering, and burst detection of keywords were analyzed in detail.

**Results:**

A total of 2,931 publications were identified, indicating a consistent increase in EEG research in stroke, with significant growth post-2017. The United States, China, and Germany emerged as the leading contributors, with high collaboration networks among Western institutions. Key research areas included signal processing advancements, EEG applications in seizure risk and consciousness disorder assessment, and EEG-driven rehabilitation techniques. Notably, recent studies have focused on integrating EEG with machine learning and multimodal data for more precise functional evaluations.

**Conclusion:**

The findings reveal that EEG has evolved from a diagnostic tool to a therapeutic support platform in the context of stroke care. The advent of deep learning and multimodal integration has positioned EEG for expanded applications in personalized rehabilitation. It is recommended that future studies prioritize interdisciplinary collaboration and standardized EEG methodologies in order to facilitate clinical adoption and enhance translational potential in stroke management.

## 1 Introduction

Stroke is a leading cause of disability and mortality worldwide, classified mainly into ischemic stroke and hemorrhagic stroke (1). Stroke results in localized or widespread neurological impairment, frequently accompanied by motor, cognitive (e.g., aphasia, executive dysfunction, and memory deficits), and swallowing dysfunctions (2, 3). Specifically, cognitive impairments vary depending on the lesion location and severity, with common manifestations including visuospatial neglect (4) and attention disorder (5). These deficits not only undermine patients’ functional independence but also impose long-term challenges for rehabilitation and quality of life. With the ongoing trend of population aging, the incidence of stroke is expected to continue rising, placing an even heavier burden on healthcare systems globally (6, 7). Traditional stroke diagnosis and evaluation rely heavily on imaging techniques such as magnetic resonance imaging (MRI) and computed tomography (CT), which are highly sensitive and specific in displaying structural brain abnormalities, particularly in the acute phase (8, 9). Furthermore, functional scales such as the NIH Stroke Scale and the Fugl-Meyer Assessment are employed to quantify functional deficits (10, 11). However, these methods are primarily oriented toward the detection of structural changes and may prove inadequate for the real-time monitoring of functional dynamics in stroke patients. Functional MRI (fMRI) and positron emission tomography (PET) can be used to assess changes in brain function but are often limited by high costs, restricting broader application (12, 13). In contrast, EEG is a non-invasive tool for monitoring brain function. It provides high temporal resolution data on neural activity by recording the electrophysiological activity of the brain (14). Furthermore, EEG is a straightforward and cost-effective method of data collection. EEG is particularly advantageous in the diagnosis and evaluation of stroke, offering the dual benefit of real-time monitoring of neurological changes in stroke patients and the identification of specific patterns of brain electrical activity through quantitative EEG (qEEG). This allows for the assessment of potential functional recovery and seizure risk (15). Post-stroke patients undergoing resting-state EEG often exhibit increased delta/theta power and decreased alpha/beta power, which correlate with motor and cognitive deficits. Functional connectivity analysis further reveals disrupted network topology in the affected hemisphere, reflecting impaired inter-regional communication (4, 16, 17). Post-stroke sleep architecture often shows reduced rapid-eye-movement sleep and increased sleep fragmentation, which correlates with poor recovery. Sleep spindles and slow-wave activity may serve as biomarkers for neuroplasticity (18, 19). In recent years, the application of EEG in stroke research has extended into advanced fields such as brain-computer interfaces (BCI) and neurofeedback (20, 21). BCI technology is capable of decoding EEG signals, thereby enabling stroke patients to control external devices through brain activity (20, 22). Neurofeedback training employs real-time EEG feedback to assist patients in self-regulating brain states, thereby promoting neuroplasticity and functional restoration (23). The combination of BCI and neurofeedback has demonstrated potential as a means of providing personalized training solutions for stroke rehabilitation. In light of these substantial applications, EEG research in the context of stroke has become of considerable value. This study employs bibliometric methods to conduct a systematic analysis of EEG research in stroke over the past 20 years. The analysis utilizes VOSviewer and CiteSpace to create a comprehensive knowledge map of the field, thereby uncovering the current state, key hotspots, and future trends. This knowledge map serves to inform and guide subsequent research.

## 2 Materials and methods

### 2.1 Data source and collection

**Figure 1:**
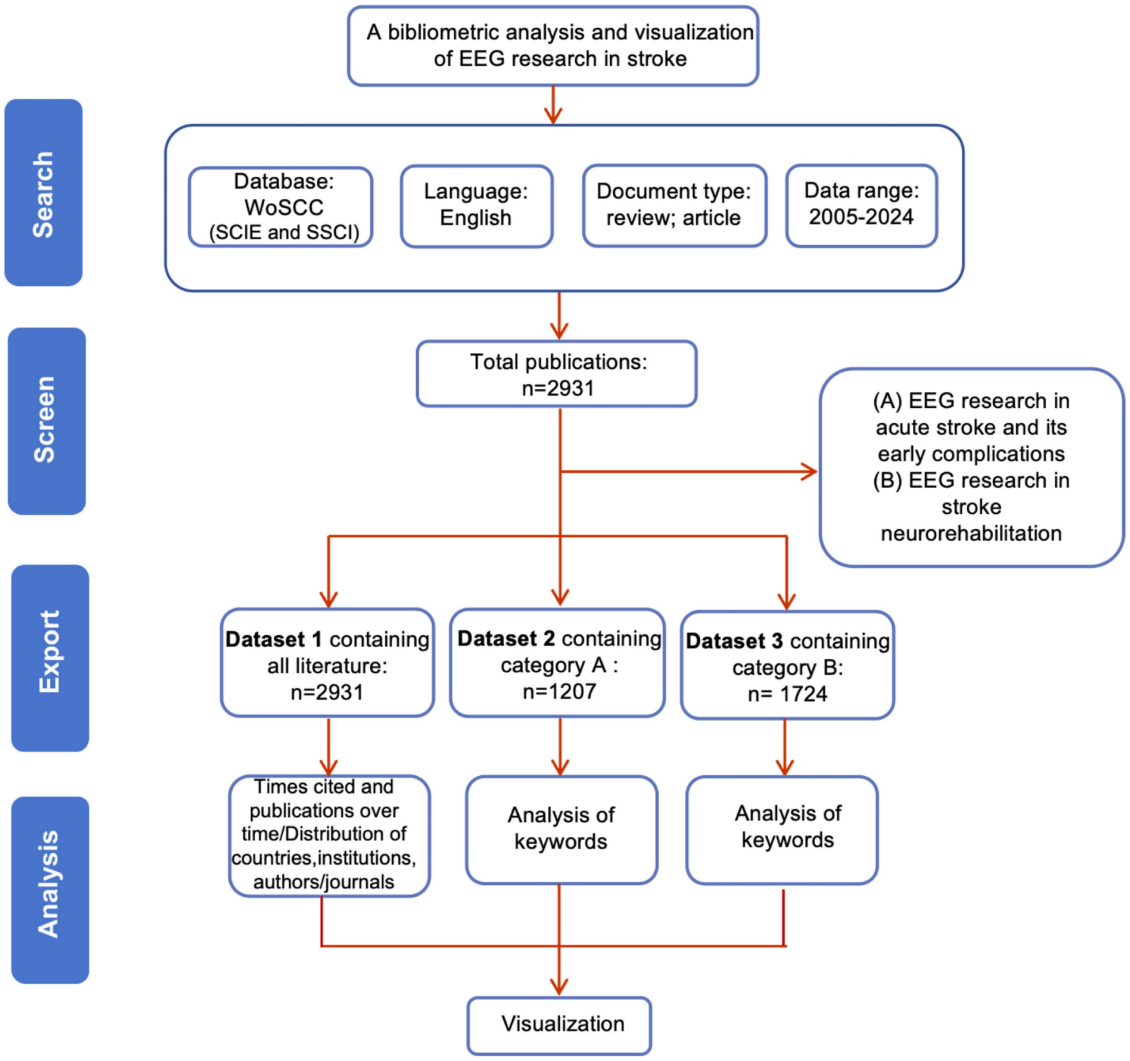
Flow chart of literature screening.

The primary data for the bibliometric analysis were obtained from the Science Citation Index Expanded (SCI-Expanded) and Social Sciences Citation Index databases (SSCI) within the Web of Science Core Collection database (WoSCC). The data retrieval strategy was summarized as follows: # 1: TS = stroke; #2: TS = (Electroencephalography OR EEG OR Electroencephalogram∗); the ultimate dataset: #1 AND #2. The utilization of a truncation symbol, “∗,” proved an effective means of preventing missed detections and enhancing retrieval efficacy. The study included only English-language studies. The time of search period was between January 1, 2005 and December 31, 2024. The search strategy is depicted in Figure 1. To minimize the potential bias from routine database updates, the literature search was conducted on a fixed date. A total of 2,931 productions were retrieved, including both reviews and research articles. To ensure the clarity and accuracy of the results and conclusions, we manually screened the 2,931 publications and categorized them into two groups: (A) “EEG in acute stroke and its early complications” and (B) “EEG in neurological rehabilitation.” Category A contained 1,207 articles, while category B included 1724 articles. The data will be stored in three separate folders: “Dataset 1” (which contains all literature), “Dataset 2” (containing only category A literature), and “Dataset 3” (containing only category B literature). Upon completion of the retrieval process, the data were saved as complete records and cited references. The articles were then extracted and exported in “Plain text file” formats.

### 2.2 Bibliometric analysis

The articles meeting the inclusion criteria were exported as a plain text file named “download_xxx.txt,” containing complete records and cited references. These files were imported into VOSviewer 1.6.19 and CiteSpace 6.2.R2 to construct visual knowledge maps. Additionally, Excel was used for chart creation and descriptive statistical analyses. The VOSviewer parameters were configured as follows: the normalization method was set to “association strength,” with minimum thresholds for countries/regions, institutions, authors and journals set at 5, 10, 7 and 10 publications, respectively. Keyword occurrence frequency was also considered, with a minimum threshold of 20. In CiteSpace, the analysis covered the period from January 2005 to December 2024, with a one-year time slice. Node types included keywords, and the g-index selection criteria were set to k = 25 per slice. The pruning options used were pathfinder, sliced networks, and merged networks, with all other settings left at their default values. In this study, we first analyzed the number of papers from countries, institutions, authors and journals based on Dataset 1 to summarize the current status of EEG research in stroke. We then used Dataset 2 and Dataset 3 to analyze keyword co-occurrence, keyword clustering, and emergent keywords, in order to identify current research hotspots and explore the frontiers and emerging trends in this field.

### 2.3 Annual publications and citations

**Figure 2:**
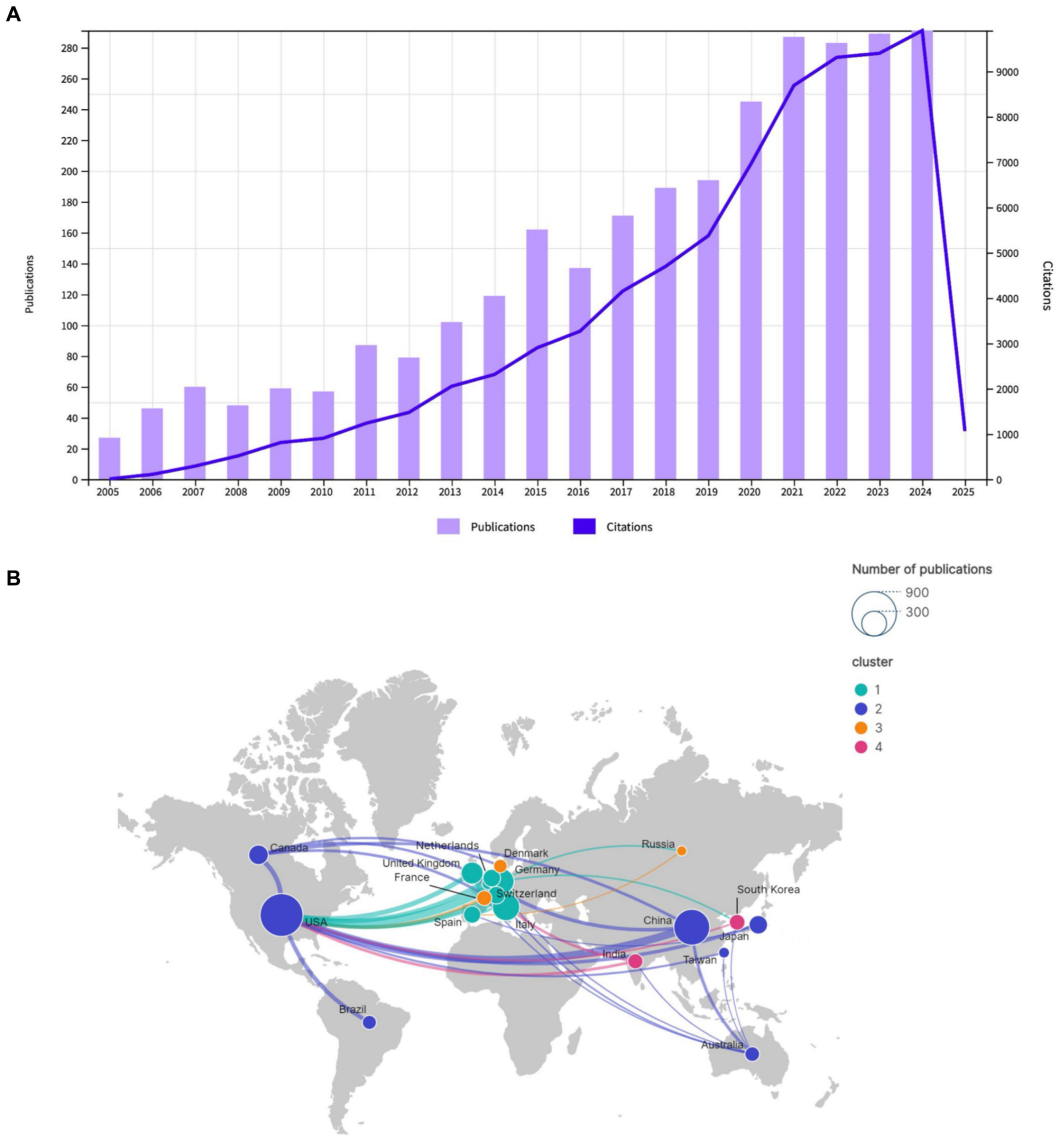
**(A)** Annual publications and citations trend chart for Dataset 1. **(B)** Geographical distribution and cooperation of publications Dataset 1.

The annual publication volume is a principal indicator for gauging research interest and predicting future dynamics in a field (24). The study encompassed 2,931 publications, comprising 2,610 original research articles (89%) and 321 review articles (11%). The total citations (TC) were 75,437. The average citation per publication (ACPP) was 25.73, and the h-index was 109. Figure 2A depicts the trajectory of annual publication volume (depicted on the left vertical axis in terms of the number of articles) and citation frequency (depicted on the right vertical axis) in the field of EEG research in stroke from 2005 to 2024. The figure illustrates an overall upward trend. The research trajectory can be delineated into two distinct phases. From 2005 to 2016, both the publication volume and the citation frequency exhibited a gradual increase. This early phase reflects the foundational work being done in the field. The moderate rise indicates a steady expansion in research and a corresponding increase in academic attention. During this period, EEG research in stroke likely laid the groundwork for more targeted clinical and experimental investigations. From 2017 onward, the field entered a phase of rapid growth, with both publication volume and citation frequency rising sharply, reaching a peak in 2024. The substantial increase in publications indicates that EEG research in stroke has gained significant traction, attracting an increasing number of researchers and funding. The rise in citations signifies a more extensive and profound integration of these studies within the broader stroke research and neurorehabilitation communities.

### 2.4 Distribution of countries/regions and institutions

A total of 2,931 publications were published by 92 countries and 3,539 institutions. A total of 57 countries and 139 institutions published at least five and ten articles, respectively, in this field. In terms of publication volume, the top five countries were the United States (778 publications), China (545 publications), Italy (316 publications), Germany (273 publications), and England (184 publications), as illustrated in Table 1. As illustrated in Figure 2B, the geographical distribution of cooperative endeavors among nations is depicted. The nodes, which represent countries, are sized according to the number of publications they have received. The upper right corner of the figure demonstrates the node sizes for publication counts of 300 and 900. The presence of lines connecting the nodes signifies cooperative interactions between countries, with the thickness of the lines denoting the frequency of collaboration. The map is color-coded into clusters and includes both the number of publications from each country as well as the strength of their collaborations. The map divides countries into four different clusters based on their collaborative relationships. Cluster 1: This cluster primarily includes countries/regions from Europe, such as Germany, England, Spain, Switzerland, Italy, and Netherlands. Cluster 2: This cluster includes countries/regions such as the United States, China, Canada, Japan, Australia, Brazil, and Taiwan. Cluster 3: This cluster includes countries/regions like France, Denmark, and Russia. Cluster 4: This cluster includes countries/regions like South Korea and India.

**Table 1:** Top 10 countries/regions ranked by number of publications.

Rank	Countries/regions	Publications	TC	ACPP	TLS	Population (million)	Publications per million people
1	United States	778	24,966	32.02	526	333	2.33
2	China	545	5,401	10.61	211	1,426	0.38
3	Italy	316	10,861	33.20	343	60.4	5.23
4	Germany	273	13,641	47.17	426	84	3.25
5	England	184	5,530	28.94	326	56	3.29
6	Canada	162	3,398	22.90	167	39	4.15
7	Japan	144	4,094	26.70	103	123	1.15
8	Netherlands	131	3,730	27.75	132	17	7.71
9	Switzerland	130	5,720	42.11	180	9	14.44
10	Spain	125	6,509	49.10	185	47	2.66

**Figure 3:**
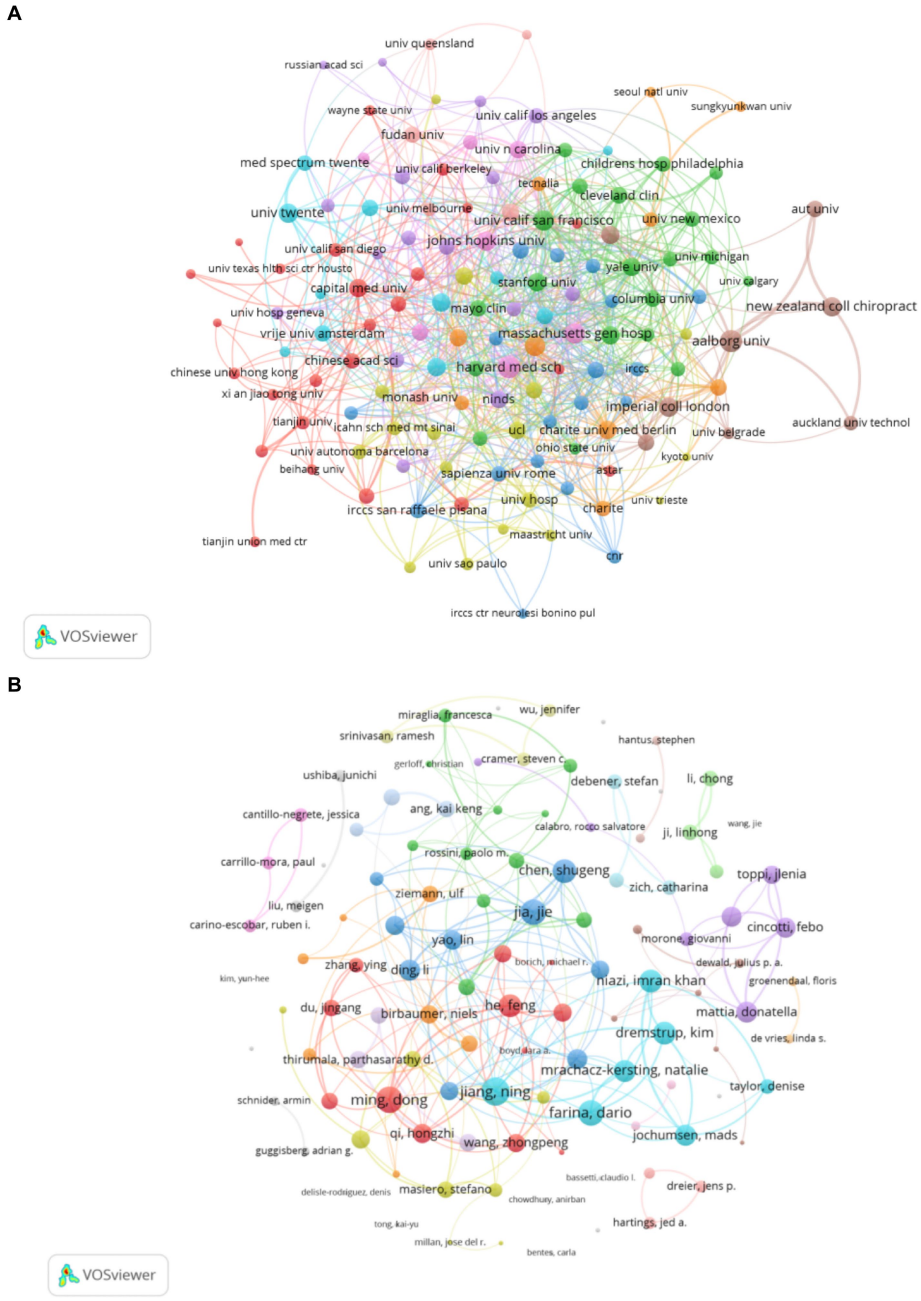
**(A)** Collaborative network knowledge map of institution Dataset 1. **(B)** Collaborative network knowledge map of author Dataset 1.

Figure 3A depicts the institutional collaboration map. Table 2 provides a detailed overview of the top institutions involved in EEG research in stroke, showing their total number of publications, TC, TLS, ACPP, and country of origin. The top 10 institutions by publication volume in EEG research in stroke show a mix of global leaders from the United States, China, and Europe, reflecting the international prominence of these universities in advancing research in this field. As illustrated in Table 2, the University of Tübingen in Germany occupies the preeminent position with 64 publications, closely followed by institutions from China, including Capital Medical University (52 publications), Fudan University (42 publications), and Shanghai Jiao Tong University (41 publications). This observation highlights the substantial contributions of Chinese institutions to the scholarly landscape. Other prominent players include Northwestern University, University of Pittsburgh, and Harvard Medical School, all from the United States, with 42, 40, and 35 publications, respectively. These institutions from the United States are widely recognized for their impactful research and often lead the field in terms of citation frequency. Notably, Aalborg University demonstrates a particularly high citation impact, with 1,205 citations, indicating that their research has gained significant academic recognition.

**Table 2:** Top 10 institutions ranked by number of publications.

Rank	Institutions	Publications	TC	ACPP	TLS	Location
1	University of Tübingen	64	4,977	77.87	51	Germany
2	Capital Medical University	52	600	11.54	37	China
3	Northwestern University	42	611	14.54	36	USA
4	Fudan University	42	656	15.62	24	China
5	Aalborg University	41	1,205	29.39	69	Denmark
6	Shanghai Jiao Tong University	41	706	19.69	31	China
7	University of Pittsburgh	40	677	16.93	27	USA
8	Tianjin University	39	510	13.08	12	China
9	Harvard Medical School	35	539	15.37	63	USA
10	University of Twente	34	1,489	36.97	49	Netherlands

### 2.5 Analysis of authors

A total of 13,234 authors contributed to this field between 2005 and 2024. Of these, 112 authors published at least seven articles with over 100 citations. The three most prolific authors in terms of publication volume were Birbaumer, N (29 publications), Jia, J (27 publications), and Ming, D (25 publications), as illustrated in Table 3. Professor Birbaumer, N from Germany is the most prolific researcher in the field, with the highest number of publications, TC, ACPP, and H-index. His research output is concentrated in the early period of his career, making him the founder and most influential scholar in the field. Among the top 10 high-impact authors, three are from China. Their publications are concentrated in recent years, and their collaborations are limited, with close collaboration within their teams but relatively few collaborations with teams outside their institutions, particularly across borders, as shown in Figure 3B. Notably, Van Putten, MJAM, a researcher from the Netherlands, has attained a commendable ACPP score of 49.85, underscoring the substantial academic recognition of his contributions to the field of EEG research in stroke.

**Table 3:** Top 10 authors ranked by number of publications.

Rank	Author	Publications	TC	ACPP	H-index	TLS	Location
1	Birbaumer, N	29	3,268	112.34	117	27	Germany
2	Jia, J	27	480	17.78	39	52	China
3	Ming, D	25	374	14.96	20	57	China
4	Jochumsen, MR	22	555	25.23	21	38	Denmark
5	Ushiba, J	22	802	36.46	30	13	Japan
6	Jiang, N	22	1,002	21.50	37	66	China
7	Niazi, IK	21	839	39.95	29	44	New Zeeland
8	Van Putten, MJAM	20	997	49.85	47	2	Netherlands
9	Thirumala, PD	19	245	12.89	21	26	USA
10	Ziemann, U	19	631	33.21	102	22	Germany

### 2.6 Analysis of journals

**Figure 4:**
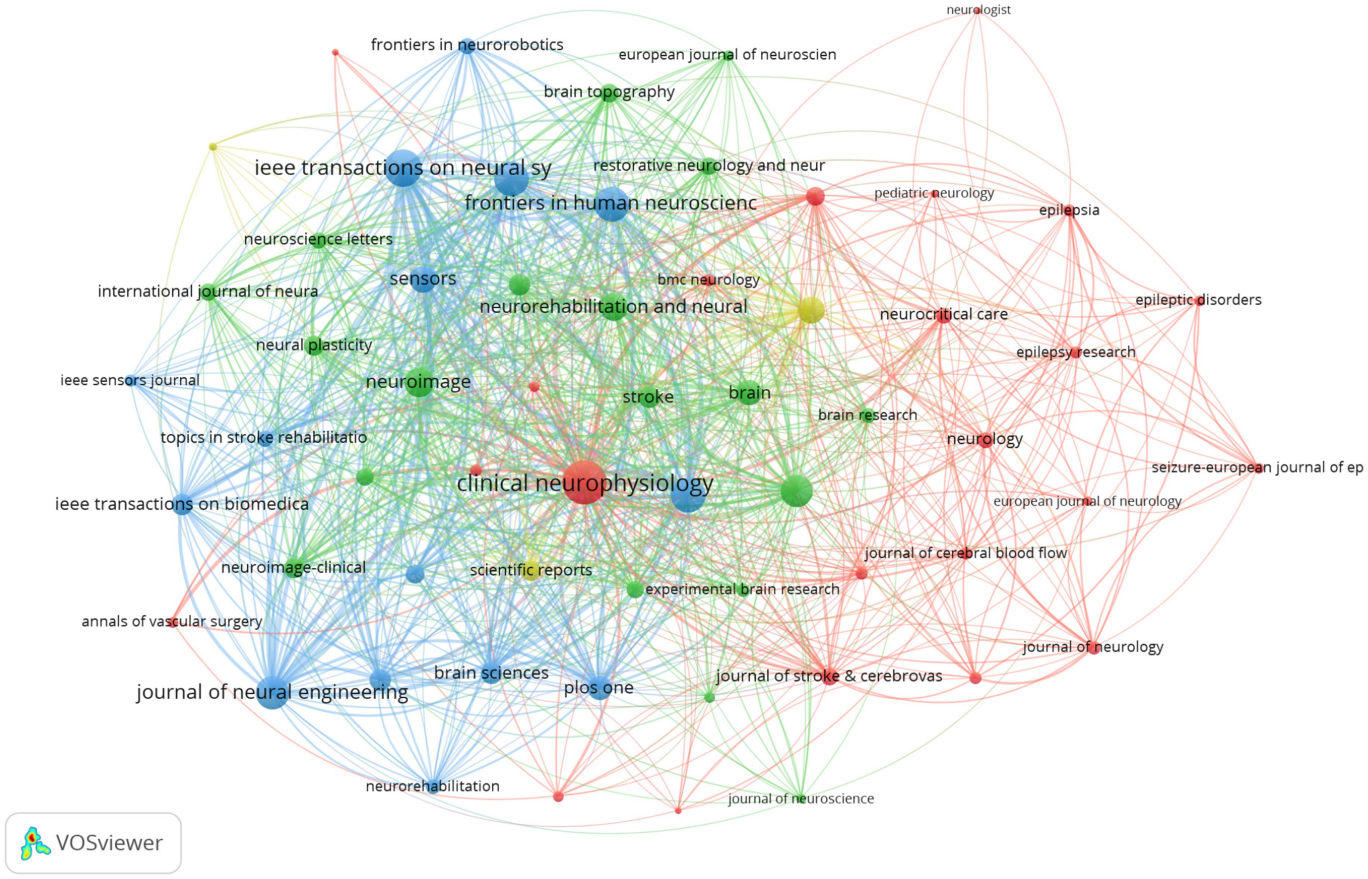
Journal co-citation network knowledge map Dataset 1.

A total of 2,931 publications were retrieved and published across 641 journals. Among the retrieved publications, 60 journals had at least 10 publications and over 100 citations, as illustrated in Figure 4. Table 4 presents the 10 journals with the highest TC. The top five journals in terms of citation frequency are *Clinical Neurophysiology* (3,191 citations), *Journal of Neural Engineering* (3,033 citations), *Neuroimage* (2,676 citations), *Frontiers in Neuroscience* (2,325 citations), and *Sensors* (2,251 citations). These journals are all ranked in the first and second quartiles by the Journal Citation Reports (JCR), which indicates that they are of high research quality and influence. Among the top 10 journals, *Brain* has the highest impact factor (IF), and despite a relatively lower publication count, it has the highest ACPP, which serves to underscore its academic prestige and broad influence in neuroscience.

**Table 4:** Top 10 journals ranked by citation frequency.

Rank	Journal	TC	Publications	ACPP	TLS	IF (2023)	JCR
1	Clinical Neurophysiology	3,191	85	35.81	963	3.7	Q1
2	Journal of Neural Engineering	3,033	74	40.75	522	3.7	Q2
3	Neuroimage	2,676	47	55.42	382	4.7	Q1
4	Frontiers in Neuroscience	2,325	78	119.94	565	3.2	Q2
5	Sensors	2,251	50	25.91	291	3.4	Q2
6	IEEE Transactions on Neural Systems and rehabilitation engineering	2,119	114	18.59	661	4.8	Q1
7	Journal of Neuroengineering and Rehabilitation	2,118	57	37.16	581	5.2	Q1
8	Brain	1945	15	129.67	261	10.6	Q1
9	Frontiers in Human Neuroscience	1828	81	22.57	563	2.4	Q2
10	Neurology	1,479	21	70.43	82	7.7	Q1

### 2.7 Analysis of keywords

#### 2.7.1 Analysis of keyword co-occurrence

**Figure 5:**
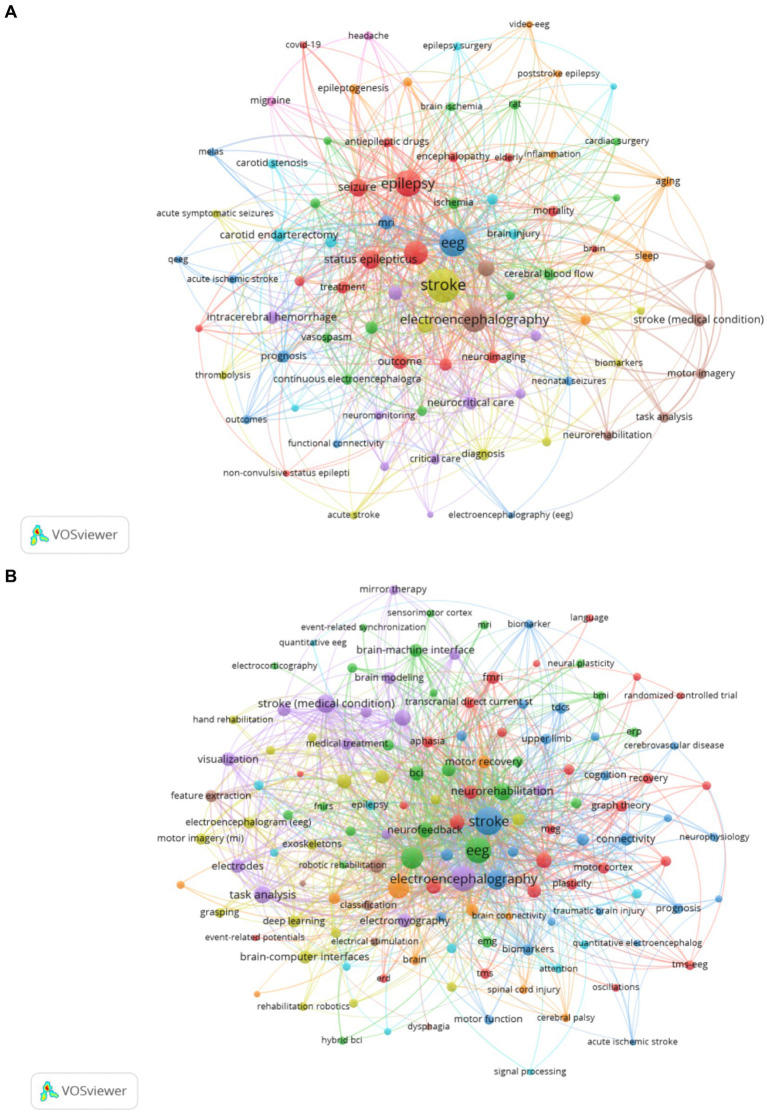
**(A)** Keyword co-occurrence network knowledge map for Dataset 2. **(B)** Keyword co-occurrence network knowledge map for Dataset 3.

Keywords are a high-level summary of the topic and content of the article. An analysis of keyword co-occurrence can reflect the hotspot and trend of research in the field (25). This study analyzes the keyword co-occurrence patterns across two distinct categories of EEG research in stroke. Table 5 shows the top 20 keywords from both Dataset 2 and Dataset 3 ranked by TLS. In Dataset 2 which includes 1,207 articles focused on “EEG in acute stroke and its early complications,” the keyword co-occurrence network (Figure 5A) highlights terms like “stroke,” “EEG,” and “epilepsy” as central nodes emphasizing the focus on EEG applications for managing acute stroke complications such as seizures and non-convulsive status epilepticus. Figure 1 showcases a total of 83 keywords with a minimum co-occurrence frequency of seven. Other significant terms such as “ischemic stroke,” “MRI,” and “intracerebral hemorrhage” reflect the intersection of EEG studies with imaging and other neurological complications. This network illustrates how EEG is used to monitor and manage the early consequences of stroke indicating the importance of EEG in understanding and addressing stroke-induced brain changes during the acute phase. Dataset 3 which includes 1,724 articles focusing on “EEG in neurological rehabilitation,” shows a different pattern in its keyword co-occurrence network (Figure 5B). This figure displays a network of 137 keywords all of which appear with a minimum frequency of seven. These central terms like “brain-computer interface (BCI),” “motor imagery (MI),” and “neurorehabilitation” are prominent reflecting the shift in focus toward using EEG in stroke recovery particularly in enhancing rehabilitation strategies through BCI systems. Keywords such as “functional connectivity,” “training,” and “rehabilitation” are tightly linked indicating the growing interest in leveraging EEG to promote motor recovery and brain plasticity in the chronic phase of stroke. Moreover terms like “virtual reality (VR)” suggest an expanding interest in integrating advanced technologies with EEG-based rehabilitation. Comparing the keyword co-occurrence networks of the two categories reveals clear differences: acute stroke research is primarily concerned with monitoring and managing immediate stroke-related complications while the rehabilitation category emphasizes long-term recovery and functional improvement through EEG-based interventions.

**Table 5:** Top 20 keywords ranked by TLS.

Rank	Keyword was analyzed by Dataset 2	Frequency	TLS	Keyword was analyzed by Dataset 3	Frequency	TLS
1	Stroke	226	352	Stroke	443	1,108
2	EEG	146	227	EEG	333	772
3	Epilepsy	112	202	Electroencephalography	261	760
4	Electroencephalography	100	187	Brain-computer interface	164	444
5	Seizures	67	152	Motor imagery	133	394
6	Status epilepticus	53	94	Rehabilitation	123	375
7	Seizure	49	84	Stroke (medical condition)	45	288
8	Electroencephalogram	38	65	Neurorehabilitation	75	247
9	Outcome	25	49	Task analysis	33	202
10	Ischemic stroke	38	47	Training	30	180
11	Traumatic brain injury	21	46	Functional connectivity	59	150
12	MRI	22	41	Neurofeedback	46	136
13	Intracerebral hemorrhage	15	40	Electroencephalogram	67	135
14	Stroke (medical condition)	11	39	Stroke rehabilitation	57	127
15	Carotid endarterectomy	40	38	Virtual reality	37	127
16	Neurocritical care	17	36	BCI	46	120
17	Magnetic resonance imaging	19	34	brain-computer interface (BCI)	57	118
18	Neuroimaging	13	33	Neuroplasticity	32	108
19	Subarachnoid hemorrhage	14	33	Transcranial magnetic stimulation	36	105
20	Prognosis	17	32	Event-related desynchronization	42	100

#### 2.7.2 Analysis of keyword clustering

**Figure 6:**
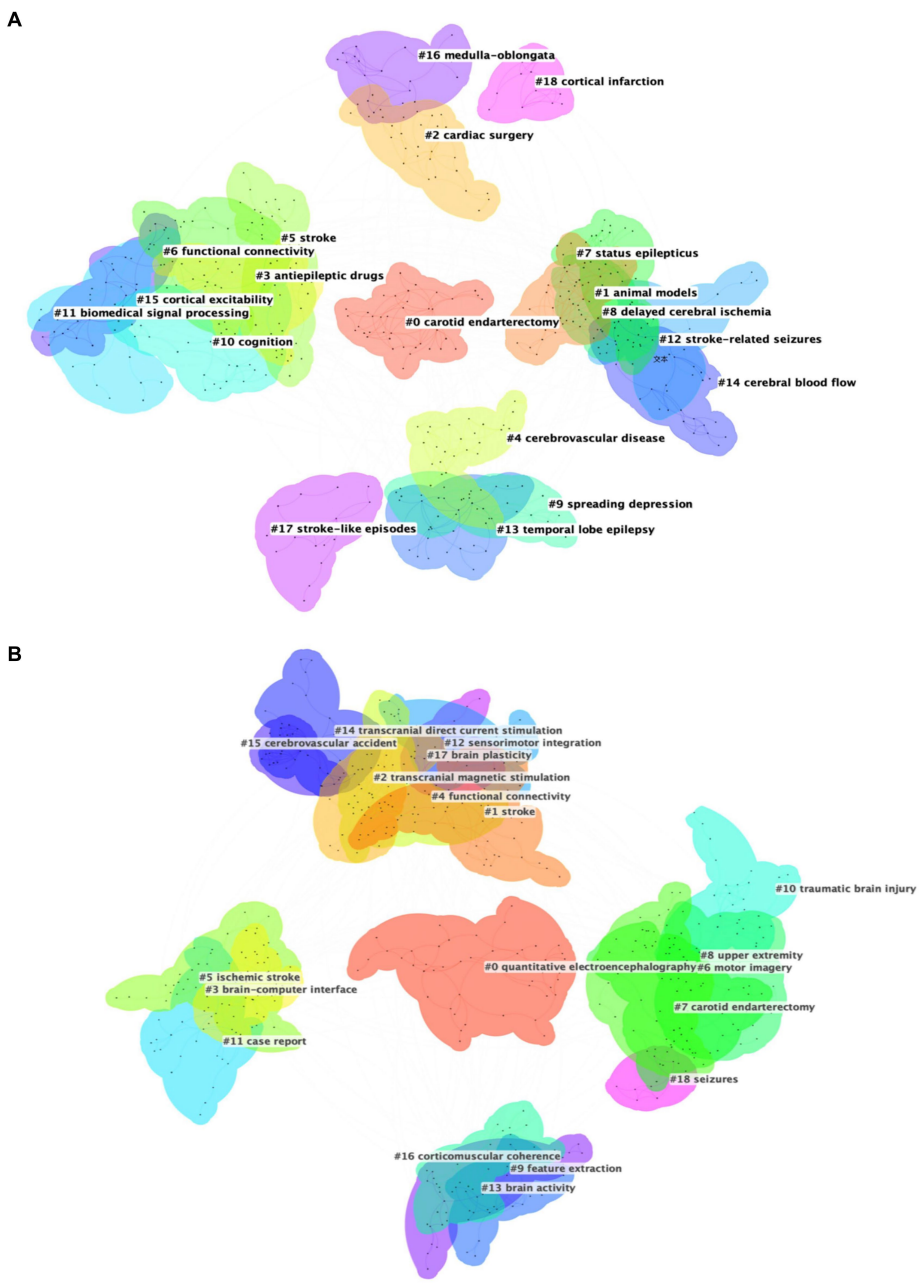
**(A)** Cluster map of keywords for Dataset 2. **(B)** Cluster map of keywords for Dataset 3.

Analysis of keywords clustering is to categorize closely related keywords, which can reveal the hotspot of research in the field (26). The collected data were imported into CiteSpace for keyword clustering analysis, the smaller the cluster number, the more keywords the cluster contains. Modularity Q is a measure of the efficacy of clustering, with a range from 0 to 1. A value approaching 1 indicates a high degree of connectivity within clusters (14). For Dataset 2, which focuses on acute stroke and its early complications, the analysis revealed a Modularity Q of 0.7094, indicating substantial network modularity and high clustering quality, as shown in Figure 6A. The LLR clustering method identified 19 distinct clusters, each representing a distinct area of research, which were subsequently labeled with descriptive terms, including #0 carotid endarterectomy, #1 animal models, #2 cardiac surgery, #3 antiepileptic drug, #4 cerebrovascular disease, #5 stroke, #6 functional connectivity, #7 status epilepticus, #8 delayed cerebral ischemia, #9 spreading depression, #10 cognition, #11 biomedical signal processing, #12 stroke-related seizures, #13 temporal lobe epilepsy, #14 cerebral blood flow, #15 cortical excitability, #16 medulla-oblongata, #17 stroke-like episodes, #18 cortical infarction. Figure 6B presents the clustering results for Dataset 3, which is centered around EEG research in neurorehabilitation of stroke, also exhibited strong clustering results with a Modularity Q of 0.7791. The LLR clustering method was employed to identify distinct 19 clusters, including #0 quantitative electroencephalography, #1 stroke, #2 transcranial magnetic stimulation, #3 brain-computer interface, #4 functional connectivity, #5 ischemic stroke, #6 motor imagery, #7 carotid endarterectomy, #8 upper extremity, #9 feature extraction, #10 traumatic brain injury, #11 case report, #12 sensorimotor integration, #13 brain activity, #14 transcranial direct current stimulation, #15 cerebrovascular accident, #16 corticomuscular coherence, #17 brain plasticity, #18 seizures.

#### 2.7.3 Analysis of keyword burst

**Figure 7:**
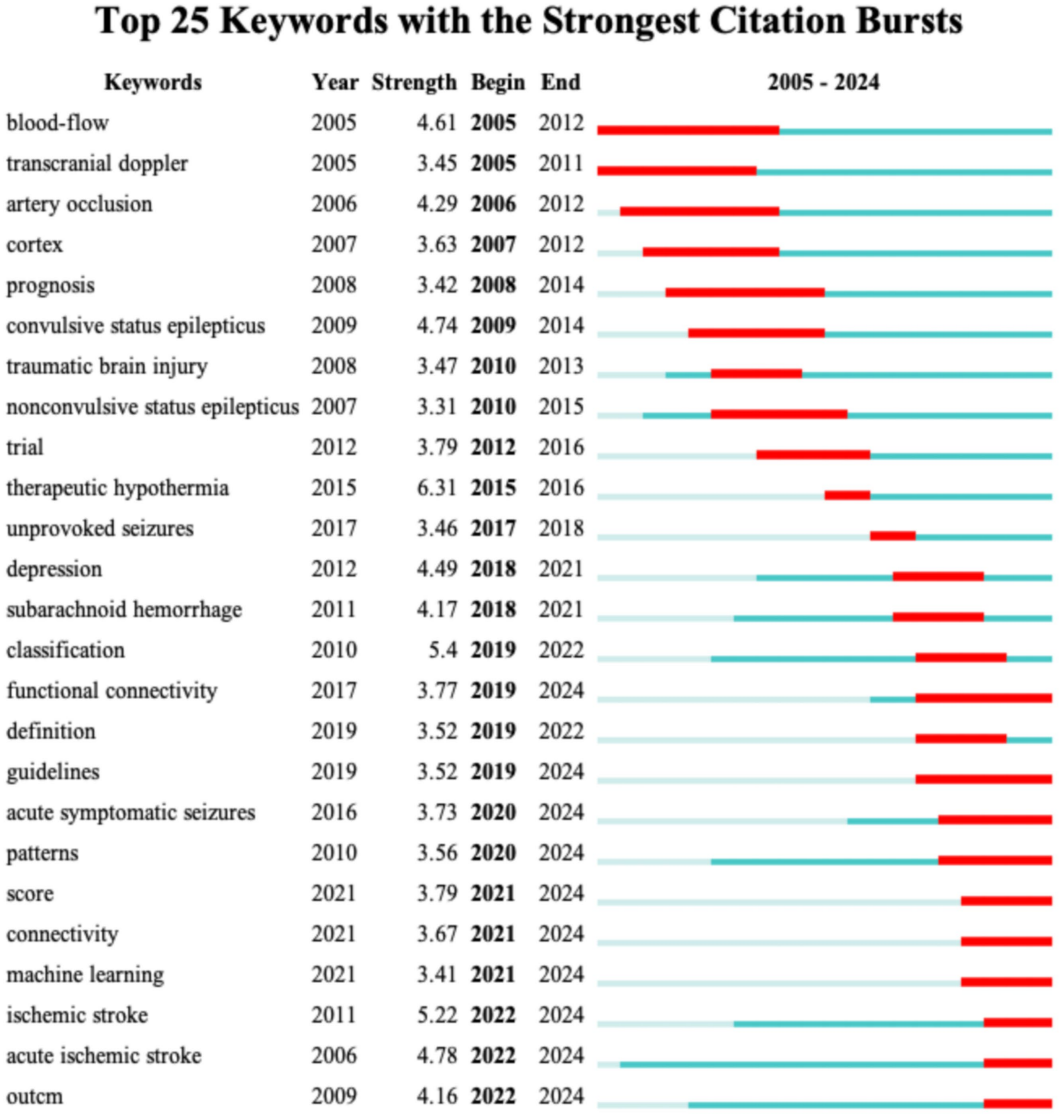
The top 25 keywords with the highest burst strength for Dataset 2.

**Figure 8:**
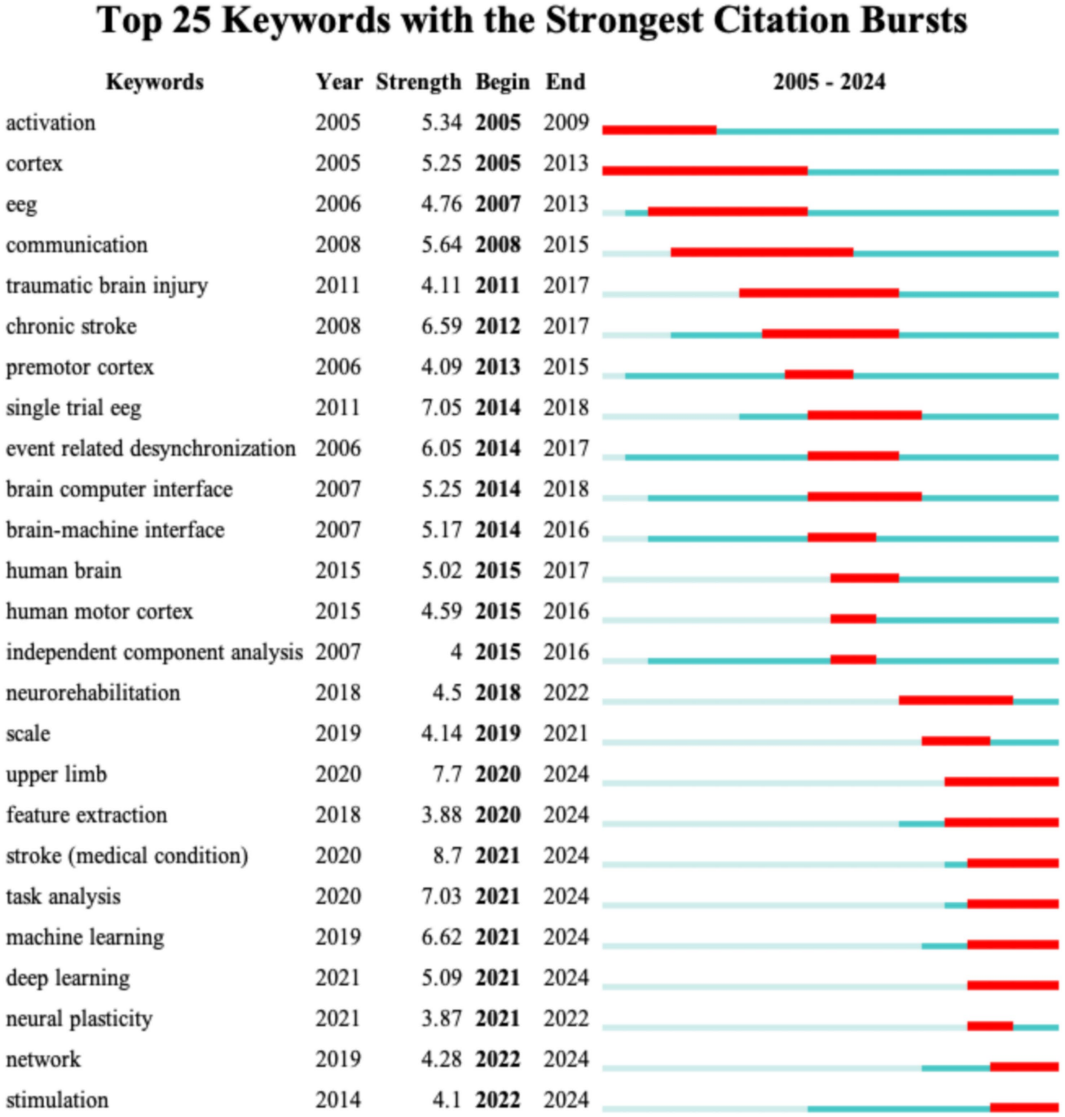
The top 25 keywords with the highest burst strength for Dataset 3.

Keyword burst analysis has been demonstrated to reveal the areas that have received the most attention within a specific timeframe thereby identifying the emerging research frontiers (26). Figure 7 illustrates the top 25 burst keywords. The “Begin” and “End” columns indicate the timeframe of the keyword burst while “strength” denotes the intensity of the burst. For Dataset 2 (analyzed with a 1-year duration parameter) Figure 7 displays the top 25 burst keywords. The earliest detected burst corresponds to “blood-flow,” which also exhibits the longest sustained burst period. Notably “therapeutic hypothermia” demonstrates the highest burst strength. The keywords “functional connectivity,” “guidelines,” “acute symptomatic seizures,” “patterns,” “score,” “connectivity,” “acute ischemic stroke,” and “outcm” are still experiencing bursts. For Dataset 3 Figure 8 reveals distinct patterns: “activation” emerges as the earliest burst keyword while “cortex” maintains the most prolonged burst duration and the keyword “task analysis” registers the strongest burst intensity. The following keywords are still experiencing bursts: “upper limb,” “feature extraction,” “stroke,” “task analysis,” “machine learning,” “deep learning,” “network,” and “stimulation.”

## 3 Discussion

### 3.1 Analysis of current research status

This study is the first to conduct a comprehensive bibliometric and visual analysis of EEG research in the field of stroke from 2005 to 2024, revealing key trends and developments. Over the past two decades, the number of publications and the frequency of citations in this area have exhibited a gradual increase. It is noteworthy that from 2000 to 2024, the annual publication volume remained above 200 publications, indicating a sustained growth trajectory in the field. This surge is indicative of the growing recognition of EEG as an essential tool in both the acute management of stroke and neurorehabilitation. The rising number of publications and the increased citation impact serve as evidence of this growing recognition.

At the national level, the analysis of publication volume and collaboration strength highlights the leading role of the United States in EEG research in stroke. Its high number of publications, citations, and collaborations with a range of countries position it as a central hub in this field. This global leadership reflects the United States’ substantial investment in neuroscience and neurotechnology, fostering an environment that supports innovation and widespread dissemination of research findings. The robust international collaboration network, particularly with countries like Germany, Italy, and England, strengthens the global impact of the United States research, accelerating scientific progress in EEG applications for stroke. China’s large volume of publications indicates a growing presence in the filed, but its relatively lower citation count and international collaboration strength suggest that its research may be more domestically focused. Countries such as Germany, Italy, and England also play pivotal roles in advancing EEG research. Germany’s high ACPP indicates that its research is impactful, shaping key innovations in the field. Meanwhile, Switzerland and Canada, though publishing fewer papers, have demonstrated strong citation impacts, reinforcing the idea that quality research can have a disproportionate effect on the global scientific community. Switzerland’s high publication count per million people further emphasizes the significant contributions of smaller nations in advancing specialized fields. As the field of EEG research in stroke continues to evolve, fostering deeper international collaboration, especially between countries with differing research capacities, will be essential for accelerating advancements.

At the institutional level, the majority of research institutions are situated in developed Western countries, with universities representing the primary contributors. This reflects the field’s reliance on economic support and experimental facilities. The University of Tübingen in Germany is the leading institution in terms of publication volume and citation frequency, exerting considerable influence, particularly in the field of BCI research. One of their most highly cited studies provides a comprehensive overview of the clinical applications of invasive and non-invasive EEG-based BCI technologies in direct brain communication and post-stroke motor recovery for paralyzed patients, demonstrating significant potential in both animal and human models (27, 28). The University of Twente in the Netherlands, despite publishing fewer papers, has a high citation frequency, indicating that their research is widely recognized for its quality. Their research has focused on qEEG technology, which has advanced the monitoring of prognosis and therapeutic responses in patients who have suffered a stroke or anoxic coma. This has established a robust foundation for the application of EEG in neuroscience (29–31). While the international collaboration network is robust (as shown in Figure 2B), our analysis shows that many research institutions collaborate predominantly with national partners (as shown in Figure 3A), with fewer instances of extensive cross-border collaboration. This suggests that geographical proximity remains an important factor influencing patterns of collaboration EEG research in stroke.

At the level of the author, Professor Birbaumer, N from Germany is one of the most prominent scholars in the field of BCI. His team has developed techniques for direct communication between the brain and external devices via EEG and other neural signals, with the objective of assisting individuals with severe paralysis and atresia syndrome to communicate with the outside world. Patients are able to control computer cursors, letter boards, or robotic arms through brain signals, thereby significantly advancing the field of BCI technology (32–34). In recent years, Professor Jia, J has been at the forefront of research utilizing BCI technology to enhance stroke rehabilitation. By integrating connectivity network patterns with spatiotemporal analysis, she has optimized EEG feature selection, thereby enhancing the efficacy of BCI applications in rehabilitation training (35, 36). Moreover, she has utilized a combination of BCI and functional electrical stimulation to markedly enhance motor function in individuals who have experienced a chronic stroke (37). Her research also encompasses traditional Chinese medicine techniques, such as electroacupuncture, and their regulatory effects on resting-state networks in stroke patients, as well as the potential clinical benefits of such techniques in rehabilitation (38). Professor Jiang N, another Chinese scholar, has also made significant contributions to the development of a single-trial detection system based on movement-related cortical potentials for BCI applications in gait initiation through extensive international collaboration (39). By interpreting patients’ motor intentions in real time through EEG signals, his work provides a basis for rehabilitative interventions for gait-impaired patients.

In terms of journal distribution, most of the journals with high publication volume and citation frequency are high-quality journals, such as *Clinical Neurophysiology*, *Neurology* and *Brain*. These journals not only provide theoretical and experimental support for EEG research in the field of stroke, but also demonstrate the academic maturity of research results in this field. As research continues to progress, future publications may increasingly be concentrated in these high-impact journals, thus creating a virtuous cycle. The platform role of these journals not only facilitates the dissemination of research findings, but also encourages further innovation within the field.

### 3.2 Analysis of research hotspots and trends

This study provides a comprehensive analysis of the keyword trends in EEG research in stroke, revealing critical insights into the evolving landscape of this field. Through the analysis of keyword co-occurrence, clustering, and burst patterns, we have identified key research hotspots, emerging trends, and shifts in the focus of EEG applications for acute stroke management and neurological rehabilitation. These findings not only highlight the current state of research but also offer directions for future investigation.

#### 3.2.1 EEG research in acute stroke and early complications

EEG research in acute stroke primarily focuses on the early identification and management of complications such as epilepsy and disorders of consciousness. The keyword co-occurrence analysis for Dataset 2 reveals a dominant emphasis on using EEG for monitoring post-stroke complications, especially seizures and non-convulsive status epilepticus, which are crucial for early intervention. Terms such as “stroke,” “EEG,” and “epilepsy” prominently feature in the research landscape, reflecting the clinical focus on utilizing EEG to understand and manage stroke-induced brain changes.

The analysis of keywords highlights epilepsy and seizures as central themes in acute stroke research. Post-stroke epilepsy is significantly associated with adverse outcomes and elevated mortality rates (40). QEEG can assist in identifying the typical EEG patterns associated with stroke (41). Non-convulsive seizures are frequently unrecognized clinically, as standard observations may prove inadequate for detecting these anomalies. Nevertheless, EEG monitoring is capable of capturing essential electrophysiological changes. Non-convulsive seizures are frequently unrecognized clinically, as standard observations may prove inadequate for detecting these anomalies (42). However, EEG monitoring can effectively capture essential electrophysiological changes. Post-stroke epilepsy in EEG typically manifests as focal or generalized slowing, with some cases also showing lateralized periodic discharges. Bentes et al. (43) conducted a study of long-term follow-up of patients who had experienced an anterior ischemic circulation stroke, finding that 25.2% experienced seizures within the first year, with 22.7% of these acute symptomatic seizures detected only by EEG. For instance, Bentes et al. (43) demonstrated that 64-channel EEG with synchronized video-polysomnography during the first post-stroke week captured electrographic seizures in over 20% of anterior ischemic stroke patients, with 62% of these events occurring during sleep. These findings emphasize the necessity of prolonged monitoring, as 22.7% of acute symptomatic seizures are identifiable only through EEG. Beyond seizure detection, EEG’s predictive capacity extends to acute neurological deficits. Vanderschelden et al. (44) conducted a prospective study of 50 acute stroke patients evaluated by recording of EEG at rest state. The delta-theta/alpha-beta ratio (DTABR) was calculated. Multivariable modeling revealed that while age, diabetes status, and infarct volume explained 47% of NIHSS score variance, adding contralesional DTABR enhanced prediction, achieving 60% explanatory power. These seizures can contribute to worsened neurological outcomes, increased risk of mortality, and prolonged recovery time, highlighting the importance of EEG in reducing these adverse effects by enabling timely treatment (45).

Expanding beyond epilepsy, EEG provides objective biomarkers for post-stroke consciousness disorders. Reduced prefrontal-to-motor cortical information flow, measurable via transcranial magnetic stimulation coupled with high-density EEG (TMS-EEG), correlates with impaired arousal states. Bai et al. (46) reported that patients with unresponsive wakefulness syndrome exhibit ≥40% reductions in gamma band connectivity between prefrontal and motor regions, while minimally conscious patients show disrupted prefrontal-parietal alpha coherence predictive of 6-month recovery. Such electrophysiological signatures align with spectral shifts observed in consciousness research: elevated low-frequency oscillations (delta/theta) and attenuated cross-frequency coupling reflect diminished cortical integration, whereas preserved theta-gamma phase-amplitude interactions may signify recovery potential (47, 48). While EEG excels in detecting electrophysiological anomalies, its integration with advanced neuroimaging techniques enables deeper insights into structure–function relationships In a targeted investigation of thalamic stroke, researchers employed DTI-derived fractional anisotropy (FA) maps alongside qEEG to probe microstructural and functional connectivity disruptions. Correlational analyses linked theta-band EEG power reductions to FA decreases in the cingulum bundle and corpus callosum-key components of the default mode network known to modulate resting-state theta activity. Alpha-band power further correlated with FA in cortico-thalamic circuits, supporting the “thalamocortical dysrhythmia” model of stroke-induced network dysfunction. This multimodal approach also bridged behavioral deficits with neural markers: FA reductions in the right cingulum predicted impaired spatial memory, while splenium of the corpus callosum correlated with facial recognition deficits (49). Thus, EEG in acute stroke research is not only focused on identifying early stroke-related complications but also on understanding the underlying neurological processes that influence stroke recovery, especially in critical conditions like seizures and consciousness disorders. While the current study primarily focuses on EEG applications in neural monitoring and rehabilitation, we acknowledge the importance of exploring broader physiological mechanisms, including inflammation and oxidative stress (ROS), in stroke pathology (50–53). Although EEG itself does not directly measure inflammatory markers or ROS levels, emerging research highlights indirect correlations between EEG patterns and these mechanisms. For example, post-stroke neuroinflammation can disrupt cortical excitability and functional connectivity, which may manifest as altered EEG spectral power or coherence (54–57). Additionally, oxidative stress has been linked to impaired neurovascular coupling (58–60), potentially affecting EEG-derived metrics. Future studies could integrate EEG with biomarkers (e.g., serum cytokines, ROS assays) to investigate these relationships. The increasing emphasis on real-time monitoring and the integration of EEG with other diagnostic tools, such as MRI or PET scans, has great potential in enhancing the clinical management of acute stroke patients, reducing mortality and improving recovery rates.

#### 3.2.2 Neurological rehabilitation in stroke

EEG research in neurological rehabilitation (Dataset 3) shifts its focus from immediate stroke complications to the long-term recovery process. The analysis of keywords for this dataset highlights the growing prominence of BCI technology, MI, and neurorehabilitation, reflecting the increased integration of EEG into rehabilitation efforts aimed at improving motor function and cognitive recovery. Terms such as functional connectivity, training, and rehabilitation emphasize the growing recognition of EEG’s potential in promoting neural plasticity during stroke recovery, particularly in enhancing motor recovery through non-invasive brain-computer technologies. One of the most profound advancements in neurological rehabilitation is the use of BCI systems. MI enables patients to engage motor-related brain regions by imagining limb movements without actual physical execution, thereby promoting neural plasticity, particularly in the recovery of upper limb and hand function. BCI technology further enhances the effectiveness of MI by decoding patients’ motor intentions and translating them into commands for external devices, thereby enabling hemiplegic patients to achieve indirect motor control (61). This not only improves motor function but also helps patients maintain active engagement during rehabilitation training. Benzy et al. (62) analyzed cortical activity during MI and successfully decoded the imagined hand movement direction (left/right) in stroke patients. The patients used the phase-locking value of EEG signals to decode the direction of imagined hand movement, which then controlled a motorized arm assistive device, allowing patients to move their impaired arms in the intended direction. The combination of EEG with EMG (electromyography) has also gained attention in recent years, particularly in enhancing the precision of motor control during rehabilitation. Li et al. (63) introduced EEG–EMG hybrid systems, which combine the advantages of both EEG for motor intention detection and EMG for muscle activity detection. This dual approach improves the accuracy of rehabilitation training, providing more personalized feedback to patients and potentially accelerating recovery. The hybrid system allows for more accurate decoding of patients’ movements and enhances their ability to perform motor tasks during rehabilitation. Another notable advancement in EEG-based rehabilitation is the application of VR in conjunction with EEG, offering patients immersive and interactive environments that stimulate motor recovery. As research progresses, the integration of EEG with VR systems is showing promise in fostering neuroplasticity by creating engaging and tailored rehabilitation experiences (64, 65). The development of signal processing technologies has greatly optimized EEG preprocessing, feature extraction, and classification methods. Traditional feature extraction frequently employs time-domain, frequency-domain, and time-frequency analyses, such as the utilization of power spectral density to examine patterns of brain activity across diverse frequency bands in patients (16, 66). The damaged regions of the brain in patients with stoke frequently exhibit augmented low-frequency bands and diminished high-frequency bands. The advent of deep learning, encompassing convolutional neural networks and generative adversarial networks, has facilitated the automated extraction of features. These neural network models allow for complex pattern recognition directly from raw EEG signals, facilitating a deeper understanding of post-stroke brain functions (67, 68). Tong et al. (69) constructed a deep learning model based on EEG signals for rapid detection of ischemic stroke. They gathered EEG data from 20 acute ischemic stroke patients and 19 healthy controls and introduced a fusion feature combining correlation-weighted phase lag index and sample entropy to explore inter-channel synchronization and functional connectivity. Recent studies have demonstrated that complex network analysis of EEG data can provide insights into the reorganization of brain networks after stroke. For example, one study (70) analyzing resting-state and task-state functional connectivity identified a “cognitive network” comprising nodes in the subcortical, frontoparietal, visual, and cerebellar networks. This network shows differential effective connectivity patterns that are sensitive to post-stroke cognitive impairment and improvement. Moreover, another study (71) focused on mild stroke patients compared EEG-based functional connectivity during cognitive tasks across groups with cortical infarctions, subcortical infarctions, and healthy controls. Their graph theory analysis revealed significantly reduced global and local efficiencies in patient groups, along with distinct nodal strength distributions that differed by lesion location. A systematic review (72) compared EEG-derived complex network parameters between stroke patients and healthy subjects. Although the effect sizes for parameters such as path length, clustering coefficient, and cohesion were modest, the review highlighted both structural differences and certain overlapping features between the groups. Additionally, multimodal data fusion techniques are increasingly applied to stroke EEG studies. By combining EEG with fMRI or near-infrared spectroscopy, researchers can obtain more comprehensive brain activity data, valuable for early prognosis prediction and evaluating the effectiveness of different rehabilitation interventions (73, 74). Stroke may not only damage local neural structures but also disrupt large-scale brain networks, affecting both structural and functional connectivity. Stroke-induced lesions may impair the integrity of the default mode network and the cortico-thalamic circuits, leading to reduced global efficiency and altered modular organization. Such disruptions may contribute to deficits in cognitive and motor function by impairing inter-hemispheric communication and reducing the integration of distributed neural systems (71, 75–77).

## 4 Limitation

Firstly, this bibliometric analysis is was confined to data drawn exclusively from the WoSCC, with the exclusion of data from other databases. This limited scope might result in the omission of some critical studies, potentially affecting the comprehensiveness of the analysis. Furthermore, the study was restricted to English-language publications, which excludes relevant research in other languages, particularly domestic studies from non-English speaking regions. This limitation could affect the representation of global research progress in the field of stroke-related EEG research. Secondly, the visual mapping generated using VOSviewer and CiteSpace required specific parameter settings, including node selection, threshold settings, and clustering methods based on data availability and study requirements. These settings may introduce some level of statistical bias, which could influence the results.

## 5 Conclusion

This study is among the first to employ bibliometric and visual analysis techniques to examine the evolution of EEG research in the field of stroke over the past two decades. The analysis was conducted using the VOSviewer and CiteSpace software tools. The results provide a systematic illustration of the current research landscape, identifying key areas of interest and future trends in this domain. The findings demonstrate that EEG is a widely utilized tool in the monitoring of neural functions associated with stroke, the assessment of epilepsy risk, and the facilitation of rehabilitation. These observations reflect a substantial academic interest and clinical relevance. The integration of deep learning and multimodal data fusion has enabled researchers to perform more complex analyses of post-stroke electrophysiological activity, laying a solid foundation for personalized rehabilitation plans. Furthermore, the use of EEG in the assessment of epilepsy and consciousness disorders improves the accuracy of post-stroke complication detection, particularly in the early identification of non-convulsive seizures and the assessment of consciousness recovery potential. In the future, as EEG technology continues to be integrated with other imaging modalities and high-efficiency algorithms, its application in stroke rehabilitation appears to be highly promising. In this context, EEG -driven BCI technologies have evolved from basic monitoring to more advanced intervention strategies. It is recommended that future research concentrate on the promotion of interdisciplinary applications of EEG and the establishment of standardized signal processing procedures. This will ensure the consistency of study outcomes and facilitate the adoption of EEG in a broader clinical context, as well as its use in translational applications.

## Data Availability

The original contributions presented in the study are included in the article/supplementary material, further inquiries can be directed to the corresponding author/s.
